# Multimodal HSI-MRI system for tumor delineation in neurosurgical guidance

**DOI:** 10.1007/s11548-026-03664-6

**Published:** 2026-04-25

**Authors:** Manuel Villa, Jaime Sancho, Gonzalo Rosa-Olmeda, Aure Enkaoua, Miguel Chavarrías, Eduardo Juarez

**Affiliations:** 1 CEIMM, Universidad Politécnica de Madrid, 28031 Madrid, Spain; 2 Department of Medical Physics and Biomedical Engineering, UCL Hawkes Institute, University College London, London, UK

**Keywords:** MRI, HSI, Multimodal, Register, Phantoms, Tumor delineation

## Abstract

**Purpose:**

This study proposes the fusion of hyperspectral imaging (HSI) and magnetic resonance imaging (MRI) for tumor delineation in image-guided neurosurgery, comparing three clinically inspired registration strategies and presenting a novel visualization for tumor resection guidance from both imaging modalities under controlled phantom conditions.

**Methods:**

A multimodal brain phantom dataset acquired with a 9.4T preclinical MRI scanner and an HSI camera was used. MRI tissues were segmented using a Segment Anything Model, while HSI data were classified with supervised XGBoost, SVM, and RF models. Three registration strategies were evaluated: surface-based registration, rigid landmark-based registration, and ArUco marker-based registration. Accuracy was assessed using fiducial registration error (FRE) and target registration error (TRE), and the overlap between modalities was quantified by the Dice similarity coefficient (DICE) and average surface distance (ASD).

**Results:**

Rigid landmark-based registration achieved the best performance (FRE of 1.7 mm, DICE of 85.4%), closely followed by the ArUco-based method, which required minimal infrastructure. Surface-based registration showed higher variability due to manual point acquisition. Overall, a high overlap between MRI and HSI tumor segmentations was observed and further enriching the information by integrating both approaches

**Conclusions:**

The proposed framework enables a reproducible evaluation of HSI-MRI fusion strategies and highlights the complementary value of volumetric MRI and surface-based HSI. Future work will focus on multimodal tissue classification, improved phantom designs, and validation with patient data.

## Introduction

Medical imaging provides noninvasive access to anatomical structures, physiological processes, and pathological alterations, while capturing the complex and heterogeneous nature of biological tissues remains challenging. Multimodal imaging addresses this limitation by integrating complementary sources of information to achieve more comprehensive tissue characterization [31], which is particularly relevant for image-guided interventions that require precise anatomical and functional information.

In neurosurgery, accurate delineation of tumor margins and preservation of healthy tissue are critical tasks due to the intricate structure of the brain and the dynamic changes that occur during surgery. Magnetic resonance imaging (MRI) is the clinical gold standard for preoperative planning, as it provides high soft tissue contrast and spatial resolution without ionizing radiation [9, 26]. However, its accuracy progressively degrades intraoperatively due to brain shift and tissue deformation [10], limiting its reliability for real-time guidance.

To address these limitations, alternative intraoperative imaging techniques such as hyperspectral imaging (HSI) have been introduced. HSI enables continuous, non-ionizing, contactless acquisition of spectral information from exposed brain tissue, capturing biochemical and physiological variations that are not observable with conventional imaging modalities [18, 27]. Despite its potential, HSI is limited by its shallow penetration depth and lack of structural context.

These limitations highlight a key challenge in image-guided neurosurgery: No single modality simultaneously provides accurate subsurface anatomical context and real-time biochemical tissue characterization, as other works suggest by proposing multimodal approaches [13, 14, 20]. This motivates the integration of MRI and HSI, where MRI contributes structural and volumetric information, and HSI provides intraoperative, tissue-specific spectral signatures. However, effectively combining these modalities requires both robust registration strategies and computational methods capable of extracting clinically relevant information.

In the case of the HSI, and due to the its high dimensionality and complexity, conventional analysis methods are often insufficient to capture subtle spectral differences between tissue types. Machine learning approaches have therefore become essential for this task. In particular, this work employs XGBoost [8], an effective framework for hyperspectral data classification, as it can model nonlinear relationships and automatically identify the most informative spectral features. For the MRI modality, a semi-automatic segmentation approach was adopted. Tumoral regions in the MRI slices were segmented using the Segment Anything Model (SAM) [16] guided by bounding boxes placed around the tumor.

Building on these approaches, the main contributions of this work are:

- Development of a novel HSI-MRI visualization system for tumor resection guidance in neurosurgery, providing a unified platform that integrates preoperative MRI and intraoperative hyperspectral data.
- Implementation and quantitative evaluation of three clinically relevant multimodal registration strategies, surface-based, landmark-based, and ArUco marker-based, using a database of brain-like phantoms, demonstrating accuracy and intraoperative feasibility.
- Integration of multimodal data fusion and analysis techniques, combining MRI-based segmentation and HSI-based classification to extract clinically relevant information beyond raw images.

## Related works

Multimodal imaging in neurosurgery is a complex topic that has received increasing interest in recent years. Most studies focus on combining multiple MRI sequences, such as T1-weighted, T2-weighted, or FLAIR, to capture complementary anatomical or pathological features [3, 12, 40]. This fusion is widely exploited in deep learning-based tumor detection and segmentation, where the inherent co-registration of MRI sequences simplifies integration and improves diagnostic accuracy.

Other approaches combine MRI with modalities such as PET, CT, or SPECT to integrate complementary anatomical and functional information [15, 38]. MRI provides high-resolution structural detail, while PET, SPECT, or CT contribute metabolic or additional structural contrast. Fusion strategies typically rely on feature or image-level integration, often designed for offline analysis, which limits intraoperative applicability.

From an intraoperative perspective, multimodal imaging has been investigated to support real-time surgical guidance in image-guided neurosurgery. Zeineldin et al. [41] proposed a multimodal neuronavigation system that integrates preoperative MRI with intraoperative ultrasound (iUS), combining real-time tracking with deep learning-based tumor segmentation and explainable artificial intelligence (AI). Their system demonstrated accurate MRI-iUS alignment and real-time performance in simulated operating room settings, highlighting the potential of multimodal integration for intraoperative guidance. Another work [19] addresses intraoperative spatial registration by leveraging point cloud representations and deep learning for surgical navigation. In this approach, medical images are converted into point clouds and registered to patient surface point clouds acquired using structured light, enabling accurate spatial alignment and augmented-reality-based visualization of the medical data.

Despite these advances, the integration of hyperspectral imaging with MRI remains largely unexplored in the context of neurosurgical imaging and guidance. Unlike conventional radiological or functional modalities, HSI provides dense spectral information related to tissue composition and biochemical properties, offering complementary intraoperative insights that are not accessible through MRI alone.

In this regard, numerous studies have explored the use of HSI to extract guidance information for brain tumor surgery [5, 24, 25, 39]. These works typically rely on machine learning techniques to classify hyperspectral signatures into different tissue types. Common approaches include support vector machines (SVM), random forests (RF), and convolutional neural networks (CNNs), among others. However, individual classifiers often face limitations in generalization and robustness when handling high-dimensional spectral data. To address this issue, ensemble-based methods have been increasingly adopted across various domains, including medical imaging, to improve classification performance by combining multiple models. In particular, methods such as XGBoost [8] have demonstrated strong performance in brain tumor classification tasks using both MRI [32, 34] and HSI data [29], as their ability to integrate multiple decision trees enables more accurate and robust tissue discrimination compared with traditional machine learning algorithms and other ensemble approaches [33].

## Materials and methods

This work presents a novel multimodal HSI-MRI visualization system for tumor delineation in neurosurgical applications. Three clinically relevant registration methodologies are employed and quantitatively evaluated using a database of preclinical multimodal brain phantoms [36, 37], enabling an assessment of the contribution of fused HSI-MRI information prior to translation to clinical scenarios.

The phantoms were designed to mimic a partial tumor resection. As a result, the phantom MRI acquisitions would be comparable to intraoperative MRI scans obtained during neurosurgical procedures. This setup enables a meaningful comparison between MRI-based workflows and HSI systems, which are more affordable and easier to integrate into the operating room environment. The following subsections describe the experimental setup, the registration strategies, and the evaluation protocol adopted in this study.

To reflect clinically relevant multimodal registration workflows, the investigated strategies are inspired by commonly used approaches in image-guided interventions.

**Method 1: Surface-based registration.** The first strategy exploits geometric features by registering a surface or point cloud acquired intraoperatively to the corresponding surface extracted from preoperative imaging. Clinically, this approach is analogous to surface tracing or facial surface registration, where a dense set of points sampled along the patient’s face is aligned with the facial surface segmented from MRI, as is commonly performed in neuronavigation systems such as the Medtronic StealthStation [21].

**Method 2: Landmark-based registration.** The second strategy is based on point-wise landmark registration, where discrete landmarks are sampled intraoperatively using a tracked pointer and matched to corresponding locations in preoperative imaging [22]. This workflow is representative of landmark-based registration procedures used in commercial neuronavigation systems, including the Medtronic StealthStation, where anatomical or artificial landmarks are manually localized to compute the image-to-patient transformation.

**Method 3: Marker-based registration.** The third strategy relies on fiducial markers directly attached to the object of interest. These markers can be detected in optical images and segmented in MRI scans due to their distinctive appearance. This approach reflects clinical marker-based registration techniques, in which optical or radiopaque markers are used to establish the spatial relationship between different imaging modalities. An example of this methodology is used in practice by companies such as Clear Guide Medical [17].

### Experimental setup

The database used in this study is based on phantoms designed to mimic the behavior of brain tissue by reproducing realistic MRI relaxation times (T1 and T2) [4, 11] with agar gel acquired with a 9.4T preclinical MRI system (MR Solutions). The dataset also includes hyperspectral images with 24 spectral bands in the range from 660 to 950 nm, acquired using a Ximea Snapshot NIR camera. Spatial tracking of the objects in the scene was performed using ten PrimeX 22 cameras (OptiTrack) and a Micron Probe (OptiTrack), which was used to sample points on predefined or mimicked soft intraoperative landmarks.

**Fig. 1:**
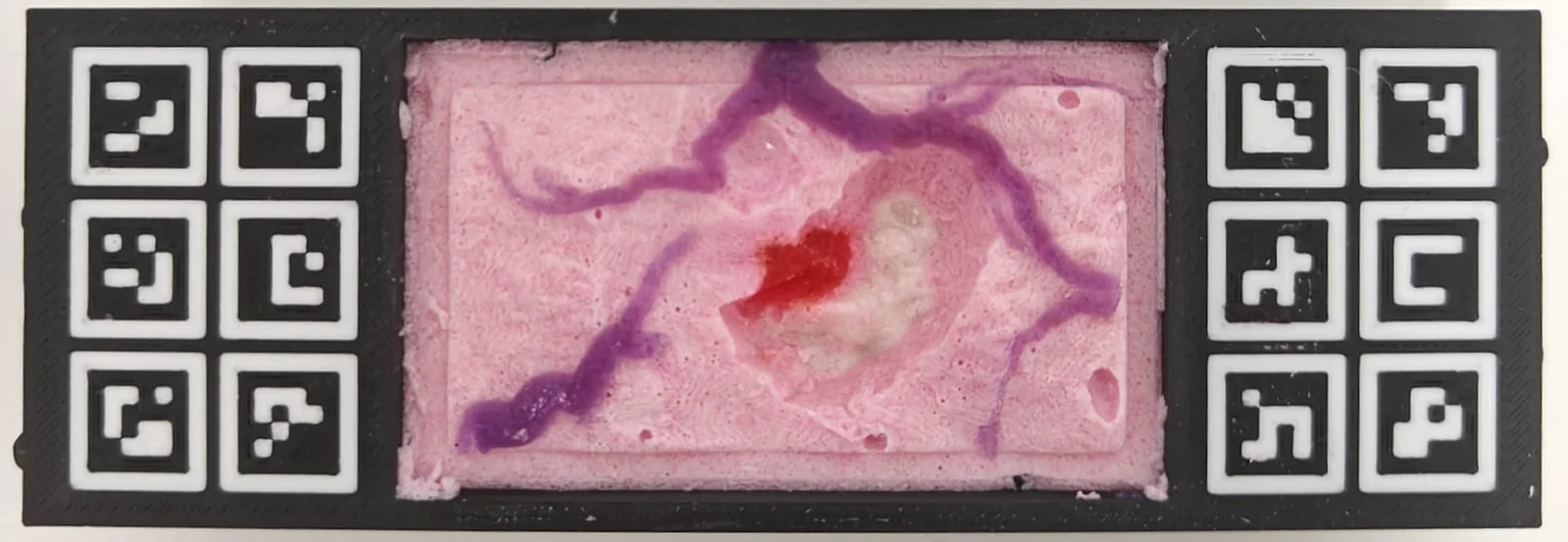
Reference image of a Phantom v2 from the database [37]. Red represents tumor tissue, white is white matter, pink is gray matter, and purple indicates blood vessels. The phantom container includes ArUco markers for registration purposes and predefined rigid landmarks

Each phantom consists of three tissue layers representing white matter, tumor, and gray matter, enabling a controlled evaluation of multimodal registration and classification [36, 37]. A partial tumor resection has been conducted on these phantoms in order to expose the tumoral area to the HSI camera and to simulate an intraoperative MRI scan. An example from the Phantom v2 dataset is shown in Fig. 1.

The use of phantoms provides the controlled conditions required for this comparison, enabling precise reference alignments before addressing realistic clinical scenarios. In addition, this setup allows for the characterization of the different error sources such as the tracking system, camera to tracking calibration, and user dependent variability associated with manual point selection. Additionally, the dedicated phantom container is designed to accommodate different registration methods.

For example, due to the limited field of view of the preclinical MRI scans used to acquire the phantom data, the integration of optical-MRI fiducial markers or rigid landmarks that are simultaneously visible in both modalities is challenging. The container incorporates optical markers printed on its surface for camera-based registration, as well as predefined rigid landmarks, while enabling MRI-to-container alignment through distinctive geometric features. This design allows the MRI volume to be reliably registered to the phantom geometry even when only a partial view of the phantom is available during the MRI acquisition.

It should be noted that direct registration between the hyperspectral camera and the MRI volume is also possible through points acquired with the tracked probe within the agar volume. This direct approach is considered in this study as one of the evaluated registration strategies, while the container-based solution provides an alternative that allows for the analysis of the other two registration strategies described.

**Fig. 2:**
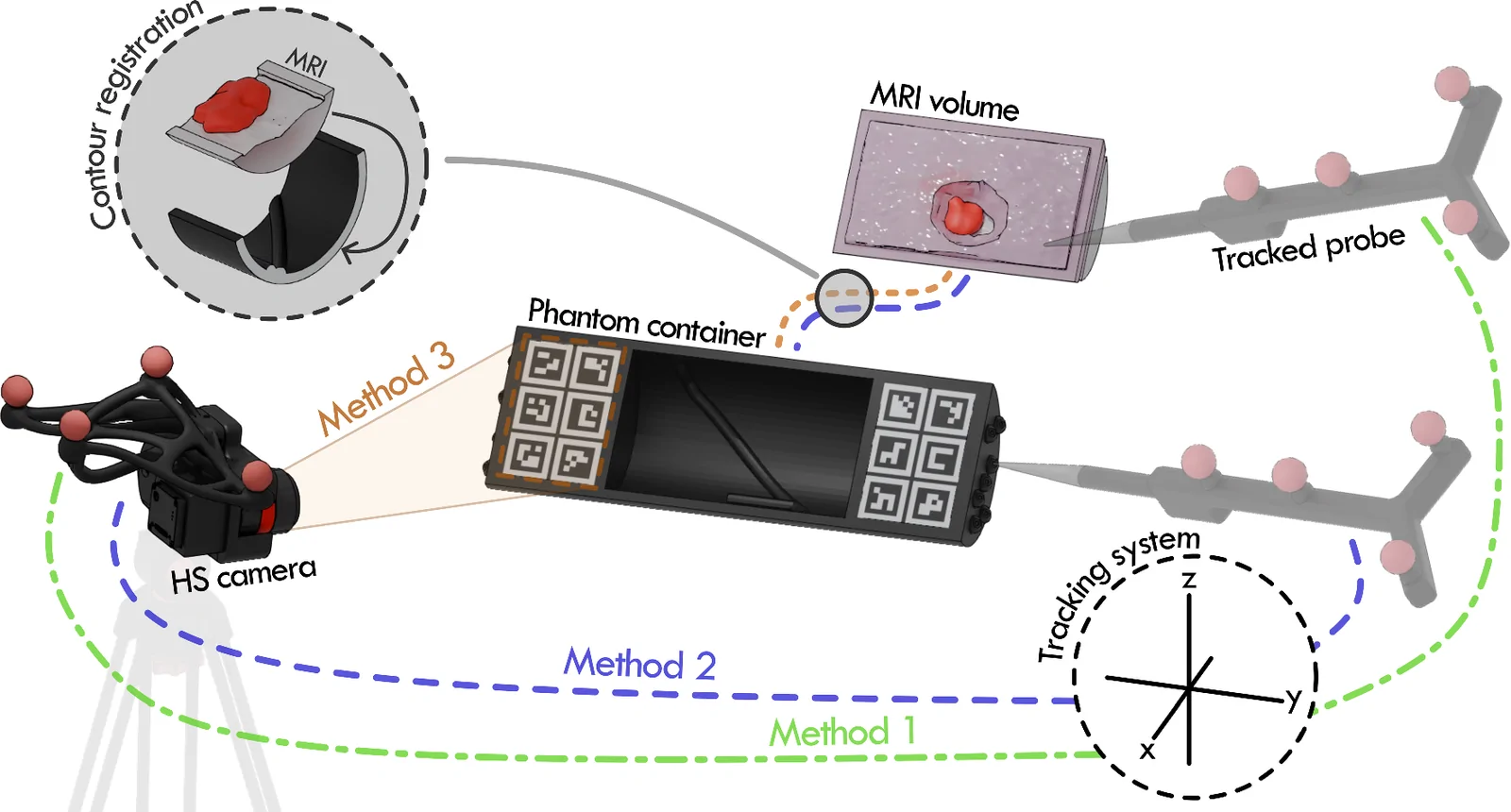
General schema of the registration methods analyzed. Method 1 (green) and Method 2 (blue) rely on the tracking system via the tracked probe and the tracking markers attached to the camera. Methods 2 and 3 (orange) use the phantom container to register the MRI volume through contour-based registration

The data fusion for these phantoms is achieved using three different methods. In Method 1, registration is performed through a common coordinate frame provided by the tracking system, where both the HSI camera and the MRI volume are independently integrated. This is illustrated by the green path in Fig. 2; in Method 2, the camera is integrated in the tracking coordinate system, shown by the blue path in Fig. 2, while the MRI volume is aligned to the phantom container geometry using contour-based registration; finally, in Method 3, the registration is based on the ArUco markers integrated into the phantom container, illustrated by the orange path in Fig. 2, where the hyperspectral camera is registered optically and the MRI is again aligned using contour-based registration. Each method is described in detail in the following subsections.

**Fig. 3:**
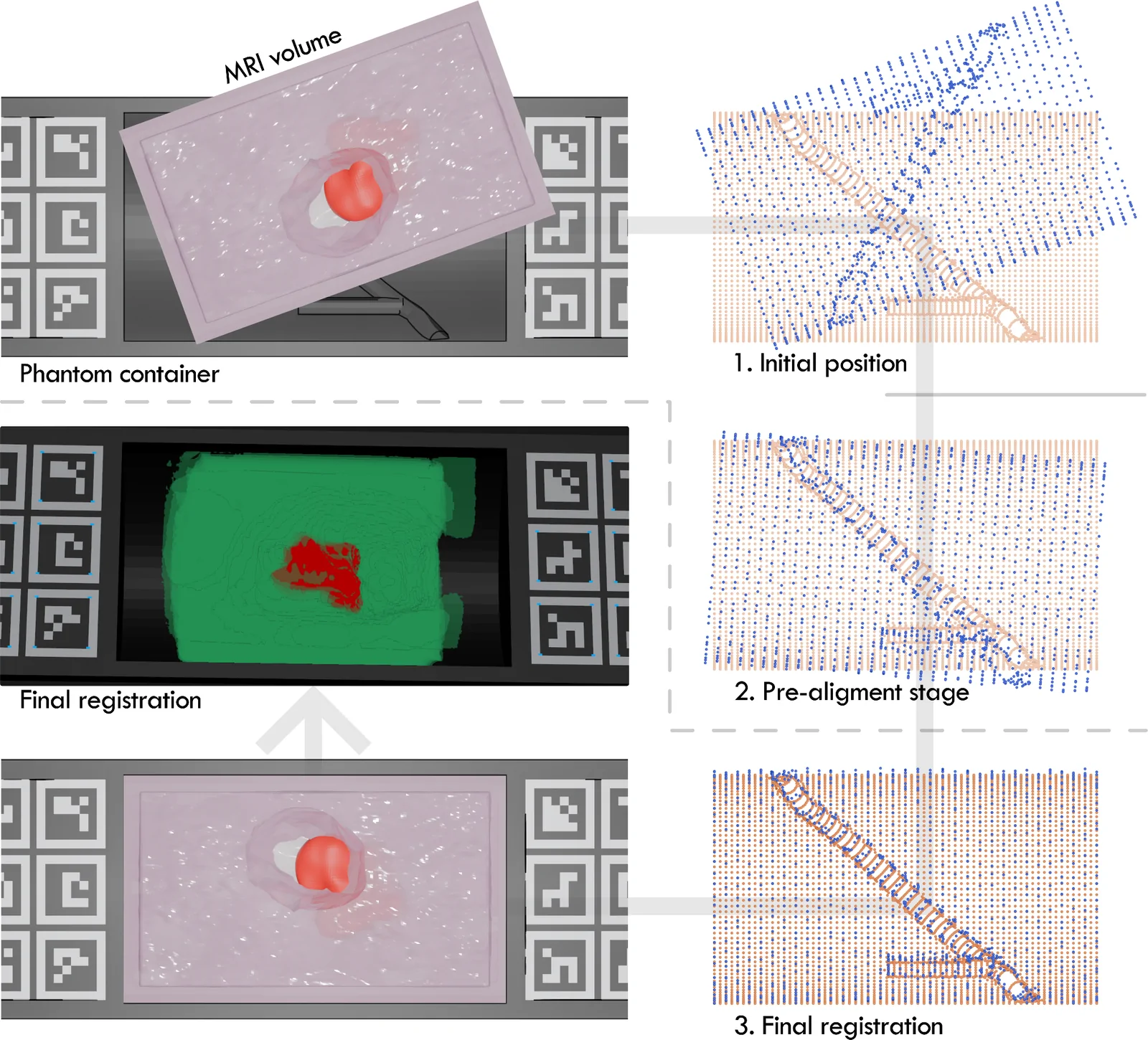
Contour-based MRI-to-container registration workflow. MRI (blue) and container contours from the 3D model (orange) are extracted (1 initial position) to compute an initial pre-alignment based on the convex regions of the diagonal inner structure (2 pre-alignment stage). The registration is then refined using all contour points (3 final registration). The final panel shows the alignment overlaid on the MRI segmentation of Phantom 4 (green: healthy tissue, red: tumor)

### Contour-based MRI-to-container registration

This registration step is essential for both the rigid landmark-based (Method 2) and optical marker-based (Method 3) strategies, as the ArUco markers and predefined rigid landmarks are defined on the container. In these cases, the MRI volume is registered to the phantom container using contour information, which is constrained by the characteristic geometric features of the container, including a diagonal inner structure and an additional orthogonal element that breaks geometric symmetry [37].

Contours are first extracted slice by slice from the MRI volume in 2D and then mapped into 3D space using the corresponding voxel coordinates. The resulting MRI contours are shown in blue in Fig. 3. The corresponding container contours (shown in orange in Fig. 3) are obtained from a voxelized version of the 3D phantom container model.

A coarse initial alignment is computed using convex regions of the extracted contours, with particular emphasis on the diagonal structure traversing the phantom volume. These regions are identified in the MRI slices using the *convexHull* function from OpenCV [6], enabling the estimation of an initial transformation that maps the MRI data into the phantom container reference frame.

As shown in Fig. 3, this pre-alignment step corrects large misorientations, including potential flips of the MRI volume. Finally, all contour points from both datasets are used as input to an Iterative Closest Point (ICP) algorithm [28], which refines the alignment and yields the final MRI-to-container registration.

### Tracking-based multimodal registration

This strategy reflects clinical workflows based on landmark registration and surface sampling, which are commonly used in neuronavigation systems.

**Fig. 4:**
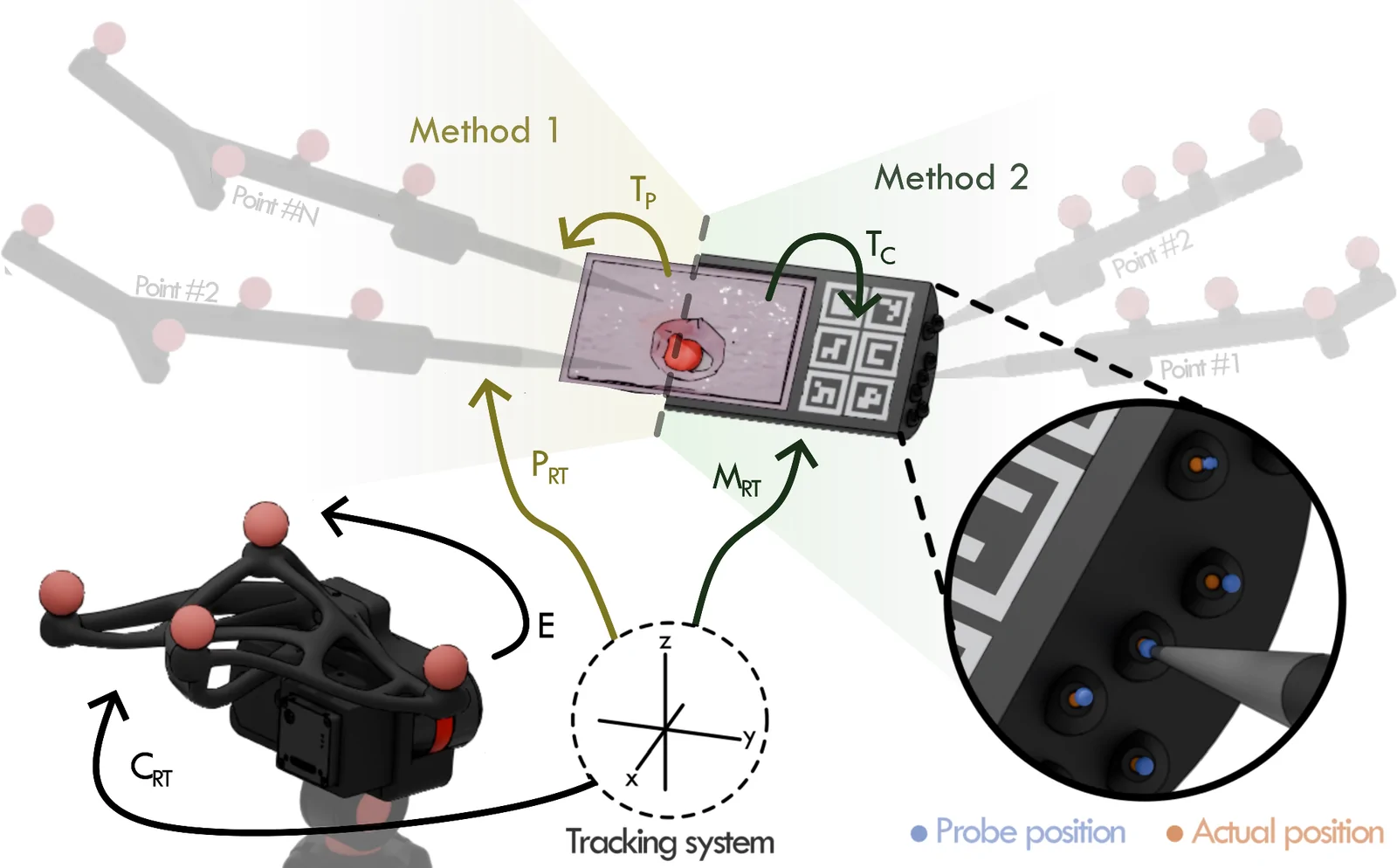
Registration workflows for Method 1 and Method 2. Method 1 (upper left, light green) performs direct registration between the MRI volume and the tracked probe ($P_{RT}$) using soft landmarks to estimate $T_P$. Method 2 (upper right, dark green) registers the phantom container to the tracking system via rigid landmarks ($M_{RT}$) and aligns the MRI to the container using contour-based registration ($T_C$). The bottom panel shows the integration of the HSI camera into the tracking coordinate system by combining the *E* and $C_{RT}$ transformations

#### Method 1: Soft landmark registration

To mimic the use of soft anatomical landmarks in a clinical scenario, such as points on the patient’s face that may deform between MRI acquisition and intraoperative localization, points were sampled directly on the phantom volume using the Micron Probe of the tracking system, as shown in the left hand of Fig. 4.

The sampled points of the MRI volume were manually localized in the MRI slices using 3D Slicer [1]. With multiple points distributed throughout the MRI volume, direct registration between the MRI and the tracking system was computed using the ICP algorithm, obtaining the transformation $T_P$ in Fig. 4 that relates the MRI points to those obtained with the tracking probe ($P_{\text {RT}}$ in Fig. 4).

**Fig. 5:**
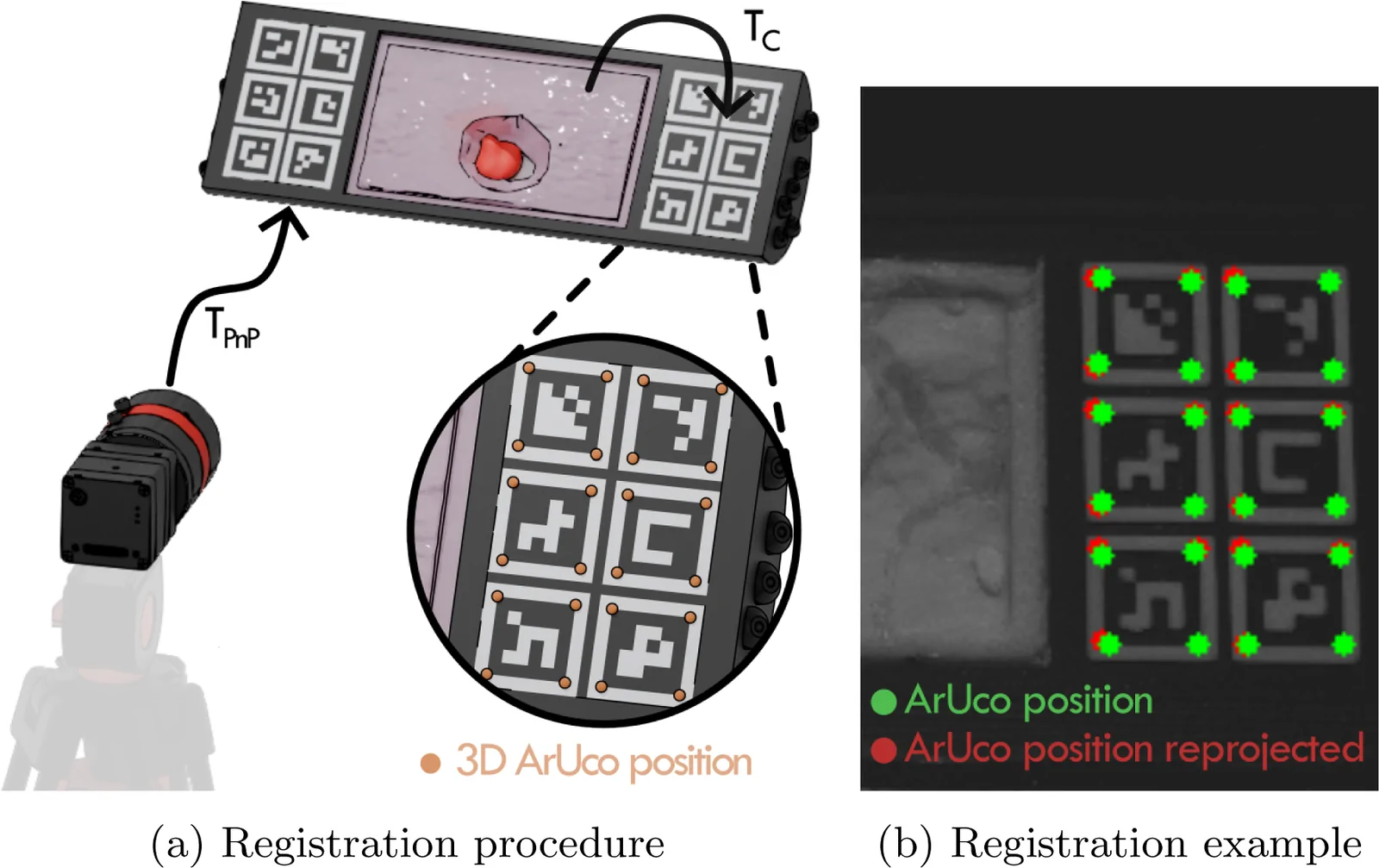
Registration workflow for Method 3. **a** Schematic of the transformations involved in the registration. **b** Example registration showing ArUco marker corners: green points represent detected corners in the HSI image, and red points are the reprojected corners using the computed transformation

Once the MRI volume was localized in the OptiTrack coordinate system, the hyperspectral camera was also integrated into this coordinate frame (transformation $C_{\text {RT}}$ in Fig. 4) following a calibration step between the camera and the tracking system. An adapted version of a previously proposed method for integrating RGB cameras into a tracking system [35] was developed for this purpose. In this case, the hyperspectral camera was directly integrated into the tracking system rather than relying on reprojection from a rigidly attached depth camera.

Due to the limited spatial resolution and the low depth of field of the hyperspectral camera, the use of a large checkerboard was not feasible. Instead, a ChessArUco board combined with a rigid set of reflective markers was employed. This configuration allowed for partial visibility of the calibration pattern among captures, which was sufficient for estimating the camera pose. The resulting calibration matrix relates the optical center of the hyperspectral camera to the tracked marker set and corresponds to matrix *E* in Fig. 4. Finally, once both the MRI and the hyperspectral camera were expressed in the same coordinate system, multimodal registration was achieved using Eq. 1.

1 $$\begin{aligned} M_{\text {MRI}\rightarrow \text {HSI}} = T_P\cdot C_{\text {RT}} \cdot E^{-1} \end{aligned}$$

#### Method 2: Rigid landmark registration

Regarding the use of rigid landmarks as bone fiducial markers, the phantom container includes predefined markers that enable this registration. Unlike Method 1, in this approach, the phantom container is first registered to the tracking system using the rigid landmarks, and the MRI volume is not directly registered to the tracking coordinate system. The landmarks are manually touched with the tracked probe (blue points in Fig. 4), while their corresponding reference positions are known from the 3D phantom model (orange points). The ICP algorithm is then applied between the tracked points and the model points to compute a rigid transformation that maps the phantom container into the tracking coordinate system, obtaining the $M_{\text {RT}}$ transformation in Fig. 4.

In parallel, the hyperspectral camera is integrated into the tracking coordinate frame using the same calibration procedure as described in Method 1. Meanwhile, the MRI volume is first aligned to the phantom container using the contour-based registration approach described in Section Contour-based MRI-to-container registration.

Once these steps are completed, the hyperspectral camera resides in the tracking coordinate frame ($C_{\text {RT}}$ and *E* in Fig. 4), the MRI is aligned to the container via contour registration ($T_{C}$ in Fig. 4), and the container is integrated into the tracking system using the rigid landmarks ($M_{\text {RT}}$ in Fig. 4). This produces the final multimodal registration between the MRI and the HSI camera, expressed in Eq. 2.

2 $$\begin{aligned} M_{\text {MRI}\rightarrow \text {HSI}} = M_{\text {RT}}^{-1}\cdot C_{\text {RT}} \cdot E^{-1} \cdot T_{C} \end{aligned}$$

### Method 3: Marker-based optical registration

The optical registration method employed in this study relies exclusively on the use of ArUco markers directly printed on the phantom container, as shown in Fig. 5a) and can be associated with the corresponding ArUco detections in the hyperspectral image.

Using these 2D–3D correspondences, a Perspective n Point (PnP) algorithm [30] implemented in OpenCV [6] is applied to estimate the pose of the hyperspectral camera with respect to the phantom container ($T_{PnP}$ in Fig. 5a). The resulting transformation is evaluated by reprojecting the 3D marker positions onto the image plane and computing the reprojection error, as illustrated in Fig. 5b), as described in Section Contour-based MRI-to-container registration.

When all the alignments have been computed, to obtain the correct relative position of the MRI with respect to the HSI camera, the transformation applied is presented in Eq. 3.

3 $$\begin{aligned} M_{\text {MRI}\rightarrow \text {HSI}} = T_{PnP}\cdot T_C \end{aligned}$$

## Experimental results

The comparison of the three registration methods described in Section Materials and methods was carried out through two complementary experiments. The first experiment evaluates the performance of the registration procedure of each method, which may involve several intermediate steps, by quantifying the fiducial registration error (FRE). The second experiment assesses the final alignment between image modalities by exploiting segmented MRI data and classified HSI information to compute their spatial overlap. In this case, the Dice similarity coefficient (DICE) and the average surface distance (ASD) between the contours obtained from each modality are used. These metrics allow the characterization of the target registration error (TRE) associated with each registration strategy.

### Classification

To enable the computation of spatial overlap between image modalities, a segmentation of healthy and tumoral tissues is required for both MRI and HSI data.

For the HSI modality, and using the labels provided in the dataset, an XGBoost, SVM [23] and random forest (RF) [7] classifier was trained on the spectral data. Hyperparameter optimization was performed using Optuna [2]. These models allow for the identification of the tissue regions exposed on the phantom surface and the extraction of the tumor contour detected in the HSI images. In addition, the HSI modality enables the classification of blood vessels incorporated into the phantom surface, which are not visible in the MRI slices due to the low signal of the material used to emulate vascular structures. The classification performance for HSI is reported in Table 1, including precision, recall, and F1-score for each labeled tissue and classifier.

For the MRI modality, a semi-automatic segmentation approach was adopted. Tumoral regions in the MRI slices were segmented using the Segment Anything Model (SAM) [16], guided by bounding boxes placed around the tumor. A similar procedure was applied to segment healthy tissue by defining bounding boxes that covered the remaining tissue while excluding the tumor area. Although the dataset provides separate labels for white and gray matter in MRI, both were merged into a single healthy tissue class for this analysis. The segmentation results for the MRI data are also reported in Table 1.

**Table 1:** Segmentation results across modalities and algorithms

Algorithm	Class	Precision	Recall	F1-score
*HSI*				
XGBoost	Healthy	93.75 ± 2.20	99.70 ± 0.30	96.63 ± 1.24
	Tumor	95.58 ± 3.46	62.10 ± 12.70	74.77 ± 10.11
	Blood Vessels	95.01 ± 11.05	80.97 ± 10.02	87.38 ± 10.26
	Background	99.96 ± 0.04	100.00 ± 0.00	99.98 ± 0.02
Random Forest	Healthy	93.93 ± 2.63	98.58 ± 0.94	96.19 ± 1.80
	Tumor	83.56 ± 9.81	62.03 ± 15.48	70.78 ± 13.29
	Blood Vessels	93.67 ± 12.45	82.47 ± 8.41	87.59 ± 9.91
	Background	99.90 ± 0.09	99.96 ± 0.05	99.93 ± 0.05
SVM	Healthy	93.19 ± 2.24	99.60 ± 0.28	96.27 ± 1.25
	Tumor	94.15 ± 4.33	64.87 ± 11.75	76.47 ± 9.55
	Blood Vessels	94.36 ± 12.45	75.98 ± 12.78	84.10 ± 12.65
	Background	99.95 ± 0.04	100.00 ± 0.00	99.98 ± 0.02
*MRI*				
SAM	Healthy	92.65 ± 16.64	69.09 ± 12.04	77.03 ± 11.24
	Tumor	93.23 ± 23.29	90.38 ± 10.86	88.31 ± 22.61
	Background	92.18 ± 18.26	67.41 ± 20.21	74.46 ± 12.21

In both modalities, the tumor class is considered the objective class. High precision is achieved for tumor detection in both MRI and HSI data. However, a higher F1-score is obtained for the MRI modality, indicating a more balanced trade-off between precision and recall compared to the HSI-based classification. Regarding the HSI classifiers, XGBoost achieves a slightly higher average performance across all classes compared to SVM, while also providing a marginal improvement in precision for tumor detection. Additionally, XGBoost requires significantly less training time, making it a more efficient choice for this application.

### Registration

For each method, the TRE was quantified by evaluating the overlap and distances between contours extracted from segmented MRI and classified HSI images. The TRE metrics, reported as DICE and ASD, are shown in Table 2. These metrics depend on the segmentation quality; however, since the segmentations are identical across methods, variations in TRE primarily reflect differences in registration performance. The FRE was computed using method-specific information, as described below:

**Method 1 (soft landmark-based ICP):** A direct registration between the HSI camera and the MRI volume is possible without relying on the phantom container. Therefore, the FRE corresponds to the root mean square error (RMSE) between points acquired with the tracked probe and their corresponding locations in the MRI volume.

**Method 2 (rigid landmark-based registration):** As the rigid landmarks are not directly visible in the MRI, registration is performed via the phantom container. Two FRE metrics are reported: the RMSE between the container points in the contour-based alignment and the RMSE after ICP registration between the 3D model landmarks and the positions acquired on the container with the tracked probe.

**Method 3 (ArUco marker-based registration):** The MRI is registered to the phantom container using contour-based alignment, while the HSI camera is registered via ArUco markers. The FRE is reported as the reprojection error of the ArUco marker corners, expressed in pixels.

**Table 2:** Registration results for the evaluated methods. FRE metrics are reported for each step in the registration pipeline, while TRE metrics quantify the overlap between MRI and HSI segmentations. The number of phantoms successfully registered is also indicated

	FRE	TRE		Phantoms registered
	RMSE	DICE	ASD	
*Method 1*				
Soft landmarks ICP	2.23 ± 1.15 mm	75.90 ± 20.38 %	3.02 ± 3.54 px	7/8
*Method 2*				
Contour registration	1.69 ± 0.33 mm	85.37 ± 11.19 %	1.99 ± 1.55 px	8/8
Rigid landmarks ICP	0.29 ± 0.06 mm			
*Method 3*				
Contour registration	1.69 ± 0.33 mm	85.79 ± 13.00 %	1.93 ± 1.65 px	8/8
ArUco markers	1.09 ± 0.06 px			

Overall, Method 2, which relies on rigid landmarks and a high-end tracking system, achieves the best performance in terms of both FRE and TRE. Method 3 follows closely, with slightly lower FRE, but it requires less infrastructure since it is independent of the tracking system and only depends on the two imaging modalities. The similarity in performance between Methods 2 and 3 is explained by the use of the phantom container as an intermediate reference in both cases.

Method 1 yields slightly worse results, with an FRE higher by approximately 0.5 mm and greater variability. The resulting DICE score is also reduced by about 10%, primarily because one phantom could not be correctly registered. This is likely due to the distribution of points acquired over the phantom volume, which may not sufficiently constrain the transformation; this is a well-known limitation in point-based patient registration, where the number, distribution, and density of the points employed matter. Method 1 additionally relies on fully manual registration, where points are collected directly on the phantom and then mapped to MRI slices, whereas Methods 2 and 3 are fully or nearly fully automated.

### Fused HSI-MRI visualization

**Fig. 6:**
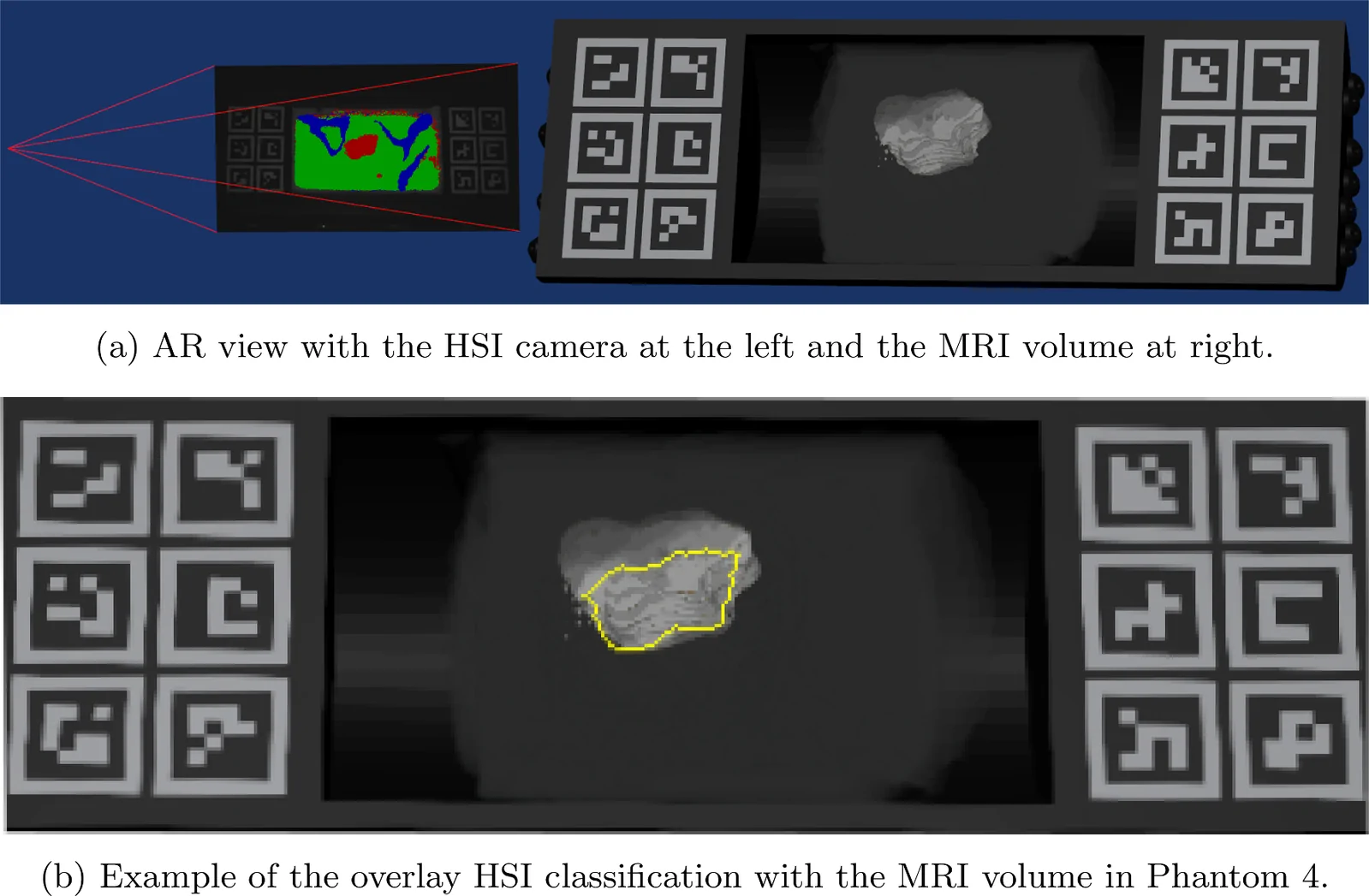
Multimodal overlay of HSI and MRI segmentations detail for Phantom 4. MRI healthy tissue is shown in semi-transparent dark gray, tumor in white, and the HSI tumor segmentation is outlined in yellow according to the applied registration transformations. Panel **a** shows an AR view with the HSI camera and 3D MRI volume, while panel **b** presents the overlap of both image modalities from the camera viewpoint

Having completed the classification and registration of the image modalities, the fused tumor delineation is illustrated in Fig. 6. In these images, the MRI segmentation is displayed in grayscale where the whitest part corresponds to the tumoral tissue and darker gray for healthy tissue, with slight transparency applied to the healthy class for visualization purposes. It is important to note that quantitative metrics are computed only on the visible surface tissues. The tumor segmentation obtained from the HSI images is overlaid as a yellow contour to facilitate visual comparison and assess the alignment between modalities across the different registration methods.

Additionally, the resulting augmented-reality (AR) visualization is shown in Fig. 6aviewpoint of the HSI camera can be overlaid onto the registered phantom, resulting in the fused visualization shown in Fig. 6b

MRI provides a three-dimensional view of the tumor, revealing subsurface regions beyond the exposed surface, while HSI captures detailed spectral information from superficial layers, enabling precise real-time delineation of tumor boundaries. Together, these modalities offer complementary guidance for both preoperative planning and intraoperative resection. For instance, in Figure 6b, the tumor visible at the surgical surface is confined within the yellow HSI-derived contours, whereas the semi-transparent MRI reveals its deeper extent, guiding a more aggressive resection in the upper region and a more conservative approach in the lower part. Relying on a single modality would omit this complementary information. While continuous intraoperative MRI could provide similar insight, it increases procedural complexity and time, whereas HSI enables real-time surface assessment without interrupting the surgical workflow and can complement the preoperative MRI information.

**Fig. 7:**
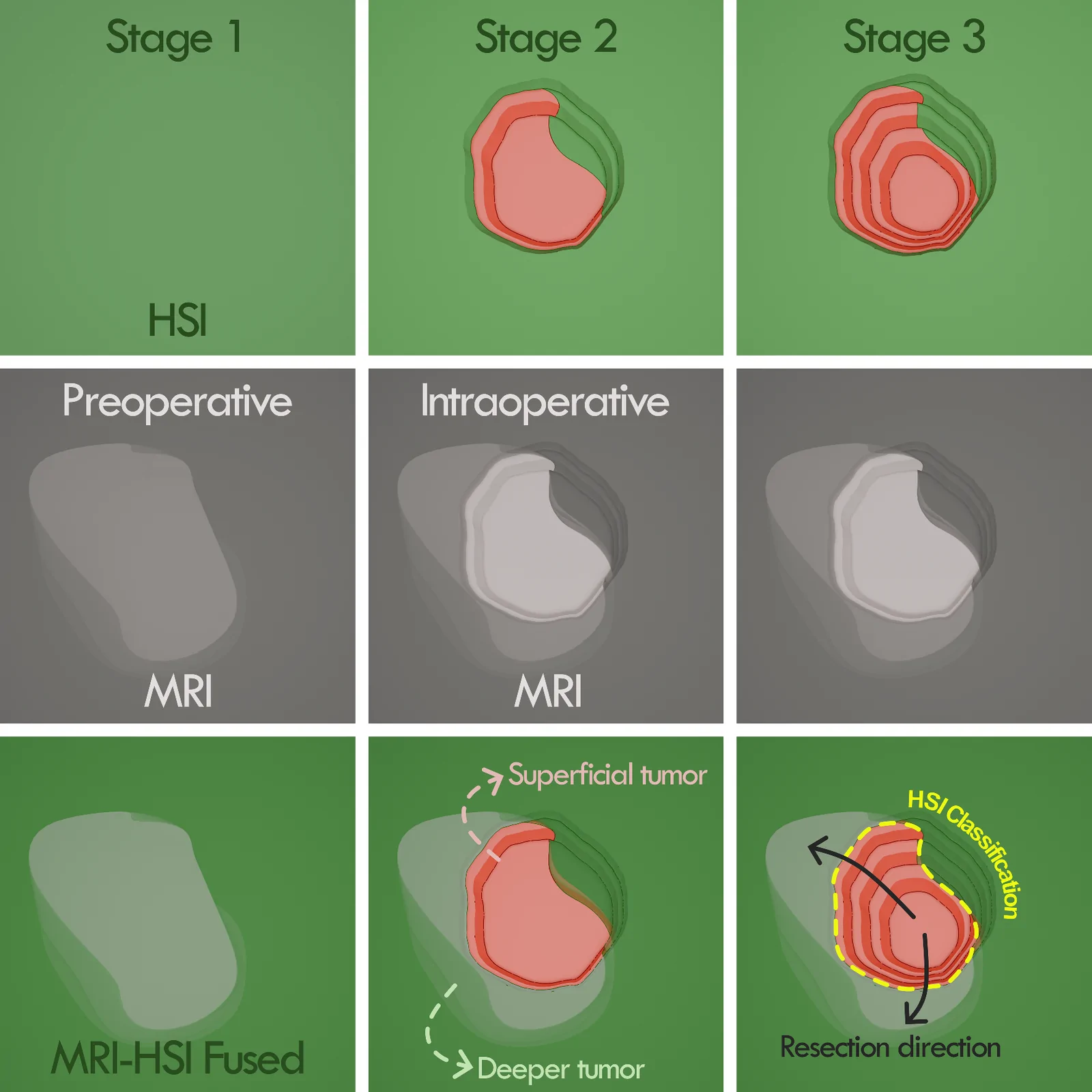
Conceptual illustration of the complementary information provided by HSI and MRI across the surgical workflow. Columns represent three surgical stages: preoperative planning (Stage 1), intraoperative exposure (Stage 2), and guided resection (Stage 3). The top row shows the HSI surface classification (tumor in red, healthy tissue in green), the middle row the MRI volumetric information, and the bottom row the fused MRI–HSI visualization, where the HSI contour (yellow) delineates the superficial tumor boundary, while MRI reveals its deeper extent, jointly guiding the resection direction

Figure 7 illustrates the conceptual motivation for fusing HSI and MRI across the surgical workflow. In the preoperative stage (Stage 1), only the MRI volume is available, providing a three-dimensional view of the tumor but without surface detail. During the intraoperative stage (Stage 2), the surgical exposure allows HSI to capture real-time spectral information from the superficial tissue layers, delineating the tumor boundary at the surface, while MRI reveals its deeper extent. In the resection stage (Stage 3), the fusion of both modalities guides the surgeon: The HSI-derived contour (yellow) delimits the superficial tumor boundary, while the MRI volume indicates the direction and depth of resection. Relying on a single modality would omit either the surface or the volumetric information, whereas their combination supports more informed intraoperative decision-making.

**Fig. 8:**
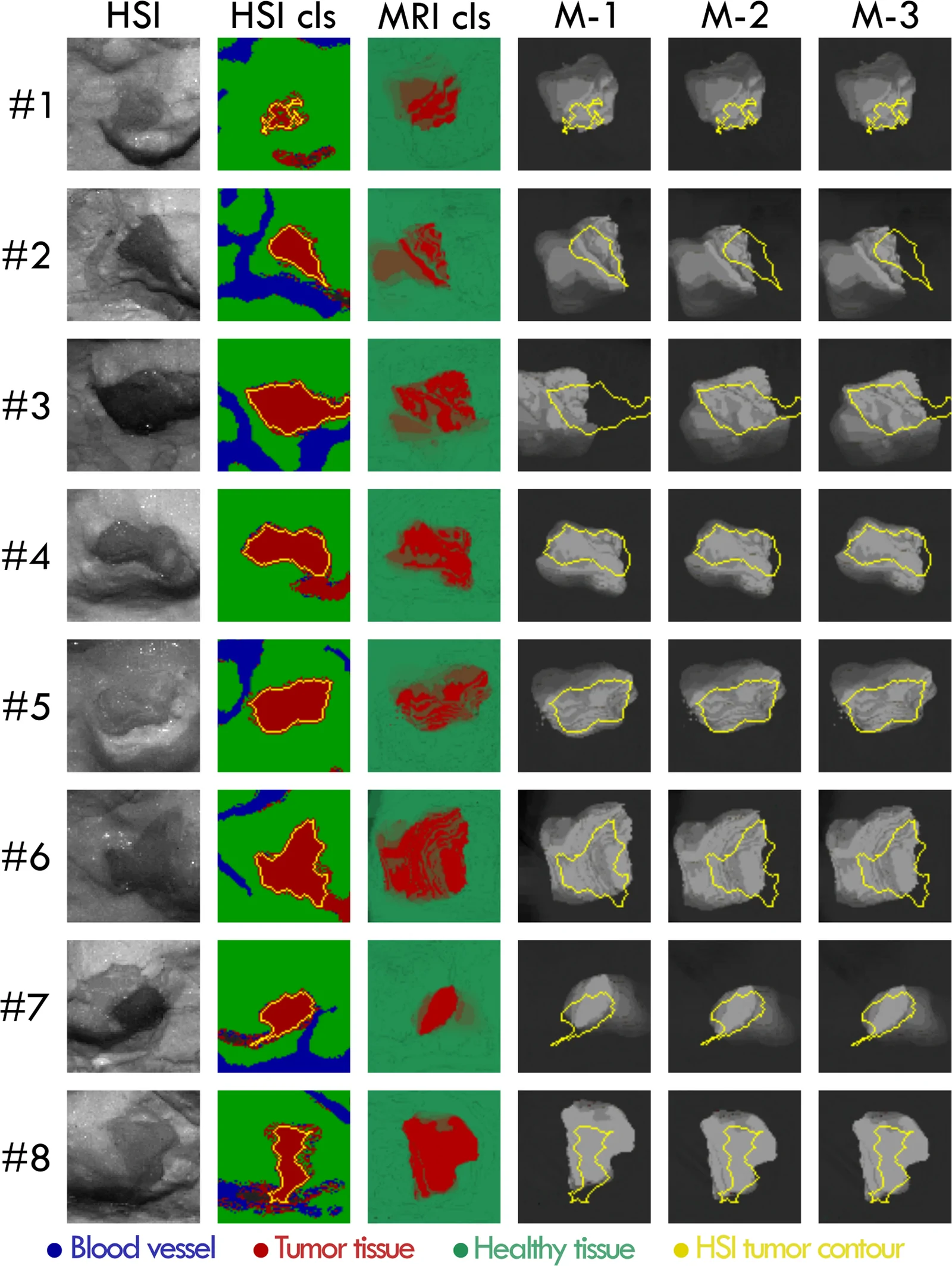
Overlap of HSI and MRI segmentations across the three registration methods for all eight phantoms. The *HSI* column shows the first spectral band (665 nm) as anatomical reference. The *HSI cls* column displays the XGBoost classification (healthy in green, tumor in red, blood vessels in blue) with the largest tumoral region outlined in yellow. The *MRI cls* column shows the SAM-based segmentation (healthy in semi-transparent green, tumor in red). The last three columns (M-1, M-2, and M-3) overlay the HSI-derived tumor contour (yellow) onto the grayscale MRI volume for each registration method. Visual discrepancies between modalities are primarily attributable to segmentation uncertainties rather than registration errors

The overall overlap between MRI and HSI segmentations across the eight phantoms and three registration methods is presented in Fig. 8. For each phantom, the independent classification results of both modalities are shown separately, HSI classification in the *HSI cls* column and MRI segmentation in the *MRI cls* column, prior to their fusion. The last three columns (M-1, M-2, and M-3) show the HSI-derived tumor contour (yellow) overlaid onto the grayscale MRI volume according to each registration transformation, enabling direct visual assessment of the spatial alignment between modalities.

The discrepancies observed between the yellow contours (HSI) and the MRI tumor volumes are mainly attributable to segmentation limitations inherent to each modality rather than to registration errors, except in specific cases (Phantom 3 with Method 1 and Phantom 2 with Methods 2 and 3). The spatial resolution of MRI limits the accurate delineation of superficial tissue layers above the tumor in the three-dimensional volume. Consequently, when the tumor is close to the surface (e.g., Phantom 6), MRI segmentation becomes more challenging and prone to inaccuracies, as reflected in the reported metrics with an increased number of false positives. Similarly, HSI segmentation may exhibit local imprecisions at tissue interfaces (e.g., Phantoms 3 and 8). Nevertheless, the proposed methodology highlights that combining both modalities enables more reliable decision-making, as their complementary spatial and spectral information helps mitigate the individual limitations of each modality.

## Conclusion and future work

In this study, we presented the fusion of MRI and HSI segmentation results using three different registration methods in a controlled phantom scenario, to provide novel visualization tools for tumor resection surgeries. The analyzed approaches reflect clinically relevant strategies for multimodal registration: Method 1, surface-based registration; Method 2, rigid landmark-based registration; and Method 3, computer vision-based registration using ArUco markers. Method 2 achieved the highest registration accuracy in terms of both FRE and TRE, followed closely by the ArUco marker-based approach. Although Method 3 offers a significantly simpler experimental setup for multimodal registration in this controlled scenario, further studies are required to assess its robustness and effectiveness in real clinical conditions, where ArUco markers would be attached directly to the patient. The surface-based method (Method 1) exhibited slightly lower accuracy and higher variability, largely due to the distribution of manually acquired points, highlighting the importance of careful point selection in clinical-like registration workflows.

The study also demonstrates the complementary nature of HSI and MRI. MRI provides volumetric context of the tumor, including underlying layers, whereas HSI offers surface information that can enhance intraoperative delineation of tumoral tissue. The integration of both modalities could support more precise tumor resection and improved intraoperative guidance. The use of phantoms and the methodology described in this work provides a controlled framework to explore multimodal imaging strategies, including potential applications in augmented-reality systems that leverage both volumetric and surface image information.

Future work will focus on applying multimodal fusion for tissue classification, exploiting the complementary information from HSI and MRI. Further studies will include phantoms designed to incorporate fiducial markers that are visible in both modalities, allowing the evaluation of registration approaches with larger MRI volumes. Ultimately, the methodology will be extended to real patient data, assessing the feasibility and benefits of multimodal HSI-MRI integration in clinical scenarios.

Overall, the presented dataset, methodology, and evaluation establish a controlled and reproducible environment for testing multimodal imaging and registration approaches, laying the foundation for future developments in image-guided neurosurgery and multimodal tissue classification.
